# Functional capacity, physical activity and muscle strength assessment of individuals with non-small cell lung cancer: a systematic review of instruments and their measurement properties

**DOI:** 10.1186/1471-2407-13-135

**Published:** 2013-03-20

**Authors:** Catherine L Granger, Christine F McDonald, Selina M Parry, Cristino C Oliveira, Linda Denehy

**Affiliations:** 1 Department of Physiotherapy, School of Health Sciences, The University of Melbourne, Melbourne, Victoria, Australia; 2 Institute for Breathing and Sleep, Melbourne, Victoria, Australia; 3 Department of Respiratory and Sleep Medicine, Austin Health, Melbourne, Victoria, Australia; 4 Department of Physiotherapy, Austin Health, Melbourne, Victoria, Australia

**Keywords:** NSCLC, Functional capacity, Strength, Physical activity, Measurement properties, Systematic review

## Abstract

**Background:**

The measurement properties of instruments used to assess functional capacity, physical activity and muscle strength in participants with non-small cell lung cancer (NSCLC) have not been systematically reviewed.

**Method:**

Objectives: To identify outcome measures used to assess these outcomes in participants with NSCLC; and to evaluate, synthesise and compare the measurement properties of the outcome measures identified. Data Sources: A systematic review of articles using electronic databases MEDLINE (1950–2012), CINAHL (1982–2012), EMBASE (1980–2012), Cochrane Library (2012), Expanded Academic ASAP (1994–2012), Health Collection Informit (1995–2012) and PEDRO (1999–2012). Additional studies were identified by searching personal files and cross referencing. Eligibility Criteria for Study Selection: Search one: studies which assessed functional capacity, physical activity or muscle strength in participants with NSCLC using non-laboratory objective tests were included. Search two: studies which evaluated a measurement property (inter- or intra-rater reliability; measurement error; criterion or construct validity; or responsiveness) in NSCLC for one of the outcome measures identified in search one. Studies published in English from 1980 were eligible. Data Extraction and Methodological Quality Assessment: data collection form was developed and data extracted. Methodological quality of studies was assessed by two independent reviewers using the 4-point COSMIN checklist.

**Results:**

Thirteen outcome measures were identified. Thirty-one studies evaluating measurement properties of the outcome measures in participants with NSCLC were included. Functional capacity was assessed using the six- and twelve-minute walk tests; incremental- and endurance-shuttle walk tests; and the stair-climbing test. Criterion validity for three of these measures was established in NSCLC but not the reliability or responsiveness. Physical activity was measured using accelerometers and pedometers. Only the construct validity for accelerometers and pedometers was reported. Muscle strength was measured using hand-held dynamometry, hand-grip dynamometry, manual muscle test, one-repetition maximum and the chair-stand test, however only two studies reported reliability and measurement error and one study reported construct validity.

**Conclusion:**

Currently there is a gap in the literature regarding the measurement properties of commonly used outcome measures in NSCLC participants, particularly reliability, measurement error and responsiveness. Further research needs to be conducted to determine the most suitable outcome measures for use in trials involving NSCLC participants.

## Background

Non-small cell lung cancer (NSCLC) is associated with significant disease burden, impaired physical status and diminished physical activity [1,2]. Due to the disease and treatment (surgery, chemotherapy and or radiotherapy) adverse physiological and psychological effects are prevalent in NSCLC, particularly exercise intolerance, weakness and impaired gas exchange and commonly a cycle of functional decline ensues [1]. Increasingly exercise interventions targeted at preventing the functional decline associated with NSCLC or improving the physical status prior to or after cancer treatment are the focus of research trials [3]. Three commonly used endpoints are *functional capacity* “the maximal capacity of an individual to perform aerobic work or maximal oxygen consumption” [4]; *physical activity* “any bodily movement produced by skeletal muscles that results in energy expenditure” [5]; and *muscle strength* “the maximum voluntary force or torque brought to bear on the environment under a given set of test conditions” [6]. The gold standard instruments (outcome measures) to assess these outcomes are laboratory based, which are not always feasible for use in research or clinical practice [7]. Therefore, a wide variety of instruments have been used to assess changes in these outcomes in the NSCLC literature.

When selecting the most appropriate outcome measure the clinician or researcher should consider the measurement properties established for their population of interest. *Reliability* determines the ability of an instrument to obtain data which are accurate, consistent and have small measurement errors when the instrument is repeated longitudinally (intra-rater reliability) or by multiple examiners (inter-rater reliability) [8,9]. *Validity* determines the ability of an instrument to measure what it is intended to measure, that is, how well the data relate to data obtained from the gold standard instrument (criterion-concurrent validity); how well data predict an outcome (criterion-predictive validity); or how well an instrument obtains data, as hypothesised, when compared to an instrument measuring a similar construct (construct validity) [8,9]. *Responsiveness* determines the ability of an instrument to detect meaningful change over time [9].Whilst a test may have excellent reliability, validity and responsiveness in one clinical population, these findings cannot always be extrapolated to other populations [9].

This review is designed to capture outcome measures applicable for use in the clinical setting by health professionals or researchers. The COnsensus-based Standards for the selection of health status Measurement INstruments (COSMIN) guidelines and the Preferred Reporting Items for Systematic Reviews and Meta-analyses (PRISMA) guidelines have been followed to report this review [8,10,11].

### Objectives

1. To identify non-laboratory outcome measures which have been used to assess functional capacity, physical activity or muscle strength in participants with NSCLC;

2. To evaluate, synthesise and compare the measurement properties established in participants with NSCLC for each of the outcome measures identified.

## Method

### Protocol

No protocol had been previously published for this review.

The search for this systematic review was conducted in two parts. Search 1 identified studies which used an outcome measure to assess functional capacity, physical activity or muscle strength in participants with NSCLC. This initial search allowed a list of outcome measures to be generated. Search 2 identified studies which examined the measurement properties of the outcome measures identified in Search 1, specifically in participants with NSCLC.

### Search 1: outcome measures

#### Eligibility criteria

##### Studies

This review considered any type of quantitative study design as defined by the National Health and Medical Research Council Classification [12]. Full manuscripts published in English in a peer reviewed journal from 1980 onwards were eligible.

##### Participants

Participants of any age, diagnosed with NSCLC, at any stage of the disease were considered. NSCLC was defined as: carcinoma of the lung including adenocarcinoma, squamous cell carcinoma and large cell carcinoma [13]. At least five participants with NSCLC were required for the study to be included. Studies which included mixed cancer cohorts were also eligible providing at least five participants were diagnosed with NSCLC. The authors were contacted for studies which did not specify the type of lung cancer to confirm the number of participants with NSCLC. Studies without original participant data (such as reviews, narratives or editorials) were excluded.

##### Outcomes

Outcomes of interest were objective tests which, based on face validity, aimed to measure functional capacity, physical activity or muscle strength in the clinical setting. Outcome measures conducted in a laboratory were excluded. Patient-reported outcome measures, such as questionnaires, were excluded.

### Information sources, search and study selection

Prior to conducting this review the Cochrane Library (including the Cochrane Database of Systematic Reviews and Database of Abstract of Review of Effectiveness DARE), Physiotherapy Evidence Database (PEDro), the COSMIN list of systematic reviews of measurement properties [14] and the International Prospective Register of Systematic Reviews (PROSPERO) [15] were searched to ensure no similar reviews had been published. Seven electronic databases were searched by one reviewer (CG) using a systematic, comprehensive and reproducible search strategy to identify all published studies (Additional file 1). Databases were accessed via The University of Melbourne and Austin Health, Australia, with the last search run on 4-October-2012.

Search terms used were: lung cancer, NSCLC, fitness, exercise, exercise capacity, functional capacity, function, acceleromet*, physical activity monitor*, global positioning system, strength, walk*, ambulat*, pedometer*, gait, outcome, assessment, test*, functional assessment, outcome assessment, exercise test, treatment outcome, data collection. A standardised eligibility assessment was performed by two independent reviewers (CG, SP) (Additional file 1). All studies identified by the search strategy were assessed based on title/abstract for eligibility. If there was insufficient information to include/exclude a study, full-text was retrieved. Consensus was required by both reviewers. Full-text of all relevant studies was obtained and read to ensure the inclusion criteria were met. Disagreements were settled by a third independent reviewer (LD). If there was insufficient information to include/exclude an article, the authors were contacted where possible. At each assessment stage agreement between reviewers was estimated with percentage agreement and the Kappa statistic using SPSS for Windows statistical software package (IBM® SPSS® Statistics Version20.0.0) [16]. All references were stored in Endnote software 2010 versionX4.

### Data collection process

A data collection form was specifically developed and used to extract data from studies by one reviewer (CG) and a second reviewer cross-checked extracted data (SP). To avoid double counting data, multiple reports on the same patient group were identified by juxtaposing study details. Collected data were stored in Microsoft^(R)^ Office Excel^(R)^2007.

### Search 2: measurement properties

#### Eligibility criteria

##### Studies

Studies which aimed to develop an outcome measure or evaluate the measurement properties of an outcome measure identified in Search 1 were eligible. Only studies published in a peer reviewed journal were included. Conference abstracts or studies not published in a peer reviewed journal were excluded due to the inability to effectively evaluate risk of bias of the individual study. Only studies published from 1 January 1980 that were available in English were eligible.

##### Participants

Participants of any age, diagnosed with NSCLC, at any stage of the disease were considered. NSCLC was defined as: carcinoma of the lung including adenocarcinoma, squamous cell carcinoma and large cell carcinoma [13]. At least five participants with NSCLC were required for the study to be included. Studies which included mixed cancer cohorts were also eligible providing at least five participants were diagnosed with NSCLC. The authors were contacted for studies which did not specify the type of lung cancer to confirm the number of participants with NSCLC. Studies without original participant data (such as reviews, narratives or editorials) were excluded.

##### Outcomes

Outcomes of interest were the measurement properties: reliability (inter- or intra-rater), measurement error, criterion validity (concurrent or predictive), construct validity (hypothesis testing) and responsiveness of outcome measures identified in Search 1 [8]. Studies validating an alternative test against an outcome measure of interest (which provide indirect evidence for validity) and longitudinal studies (which provide indirect evidence for responsiveness) were excluded because such studies have not specifically formulated or tested hypotheses about the measurement properties [8]. Studies evaluating a battery measure including a relevant sub-component were also excluded as they are designed to be used in their entirety.

### Information sources, search and data extraction

**Figure 1:**
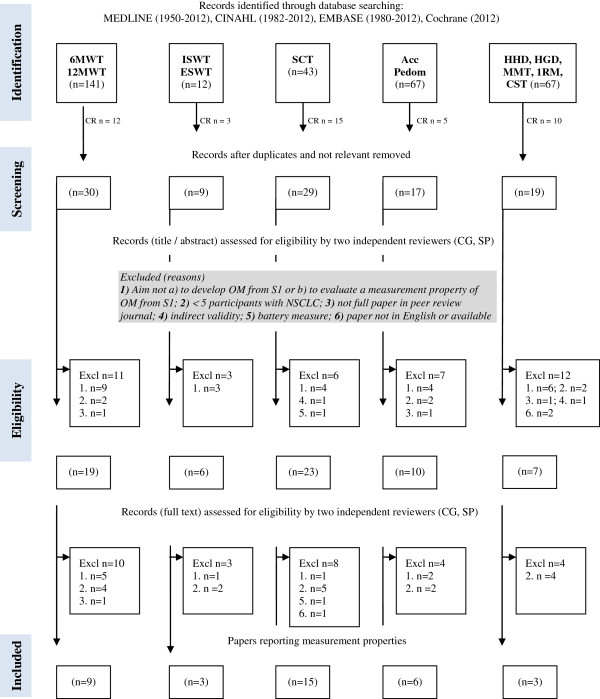
**Flow diagram of measurement properties study selection process – Search 2.** Abbreviations: 1RM, one repetition maximum; 6MWT, six-minute walk test; 12MWT, twelve-minute walk test; Acc, accelerometer; CINAHL, Cumulative Index to Nursing and Allied Health Literature; CR, cross referencing; CST, chair-stand test; ESWT, endurance-shuttle walk test; EMBASE, the Excerpta Medica Database; excl, excluded; HHD, hand-held dynamometry; HGD, hand-grip dynamometry; ISWT, incremental-shuttle walk test; MMT, manual muscle test; n, number; NSCLC, non-small cell lung cancer; OM, outcome measure; Pedom, pedometer; S1, search from part one; SCT, stair-climb test.

Four electronic databases were searched by one reviewer (CG) using a systematic, comprehensive and reproducible search strategy (Figure 1). The last search was run on 4-October-2012. A previously published search filter was used (sensitivity 97.4%; precision 4.4%) (Additional file 2) [17]. No publication date or language restrictions were imposed on the search. The study selection and data collection processes followed were the same as described for Search 1. Data items extracted were adapted from the COSMIN generalizability checklist [10].

### Risk of bias of studies

Two independent reviewers (CG, CO) evaluated risk of bias using the 4-point COSMIN checklist [18]. This checklist was originally developed to assess the methodological quality of patient-reported outcome measures however it has also been suggested for use to assess the quality of non-patient reported outcome measures [10]. Four items from the checklist (internal consistency, structural validity, cross-cultural validity and content validity) are only applicable to questionnaires and were therefore not assessed [19]. Questions for remaining items (reliability, measurement error, hypothesis testing, criterion validity and responsiveness) were scored on a 4-point scale. The overall score for each item was obtained by using the lowest score (excellent, good, fair or poor) recorded for any question within the item, as recommended by the COSMIN scoring system [18]. Reviewer agreement was estimated with percentage agreement and the Kappa statistic [16].

## Results

### Search 1: outcome measures

The search of seven electronic databases and cross referencing identified 6,398 studies. Assessment of title/abstract and full text results in 88 articles using 13 different outcome measures being included (Figure 1; Additional file 1). A list of outcome measures was generated (Table 1). Almost perfect agreement between reviewers of potentially relevant titles/abstracts (CG, SP) (97.0%, Kappa=0.93) and full-text articles (CG, SP) (94.5%, Kappa=0.82) was obtained [16]. The third reviewer (LD) was consulted twice. Twenty-two authors were contacted to clarify the cancer type, 13 responded. In ten cases the lung cancer type could not be confirmed and these studies were excluded.

**Table 1:** Synthesis of evidence regarding measurement properties: comparison of outcome measures

**Outcome measure**	**Reliability, measurement error and responsiveness**	**Criterion concurrent validity (gold standard)**	**Criterion predictive validity**	**Construct validity (hypothesis testing)**
**Functional capacity**				
**6-minute walk test**	x	x	8 studies [22-27,39-41], predicts: survival, post-op outcomes (complications, LOS, 6MWT, HRQoL) and development of RP	1 study [26,27], strongly correlated with RFT
**12-minute walk test**	x	x	x	x
**Incremental-shuttle walk test**	x	1 study [30], strongly correlated with CPET	2 studies [30,64], predicts: survival and post-op outcomes	3 studies [30,44,64], moderately correlated with muscle strength; little, moderate and strong correlation with RFT; little correlation with mastery questionnaire
**Endurance-shuttle walk test**	x	x	x	x
**Stair-climb test**	x	1 study [29], strongly correlated with CPET	13 studies [21,22,32-39,41-43], predicts: post-op outcomes (complications, LOS, cost) and survival	2 studies [20,31], strong correlation between VO_2_peak and altitude reached; direct association between pre and post-op SCT results
**Physical activity**				
**Accelerometer**	x	x	x	3 studies [46-51], strongly correlated with estimated EE and sleep; moderately correlated with depression, HRQoL and PS
**Pedometer**	x	x	x	1 study [53], moderately correlated with CPET
**Muscle strength**				
**Hand-held dynamometry**	x	x	x	x
**Hand-held dynamometry with pulley force sensor**	1 study [52], very good intra-rater reliability; large SEM	x	x	x
**Hand-grip dynamometry**	1 study [28], moderate intra-rater reliability	x	x	x
**Manual-muscle test**	x	x	x	x
**One-repetition maximum**	x	x	x	x
**Chair-stand test**	x	x	x	1 study [53], moderately correlated with PS and fatigue

Abbreviations: *6MWT*, six-minute walk test; *CPET*, cardio-pulmonary exercise testing; *EE*, energy expenditure; *HRQoL*, health related quality of life; *ICC*, intraclass correlation coefficient; *LOS*, length of hospital stay; *post-op*, post-operative; *pre-op*, pre-operative; *PS*, Performance Status; *RFT*, respiratory function tests; *RP*, radiation pneumonitis; *SCT*, stair-climb test; *SEM*, standard error of measurement; *VO*_*2*_*peak*, peak oxygen consumption.

x = not assessed.

### Search 2: measurement properties

#### Study selection

The search identified 375 studies of which 34 articles (31 studies) were included (Figure 1). Almost perfect agreement was obtained between reviewers (CG, SP) for titles/abstracts (96%, Kappa=0.92) and substantial agreement was obtained for full-text articles (90%, Kappa=0.78) [16]. Twelve authors were contacted to clarify the cancer type, nine responded. In seven cases the lung cancer type could not be confirmed and these studies were excluded.

#### Study characteristics

Table 2 summarises the 31 prospective observational studies. The majority of studies included only participants with NSCLC (n=18, 58%). Studies had a mean (standard deviation [SD]) sample size of 130 (146) participants (range 12–640). Outcome measures were longitudinally repeated in 25% of studies: before and after surgery (n=5, 16%) [20-24], chemotherapy (n=1, 3%) [25] and radiotherapy (n=2, 6%) [26-28] (Table 3).

**Table 2:** Study characteristics – part 2

**Author, yr location**	**n**	**Gender M/F**	**Age mean (SD) yrs**	**OM**	**Setting (n)% / method of pt selection**	**Cancer type (n)%**	**Cancer stage (n)%**	**Cancer treatment at baseline time-point (n)%**
**Functional capacity**								
Jones 2012 USA [40]	118	71/47	61 ± 10	6MWT	Health system / consecutive	NSCLC	IIIB, IV, recurrent IV	Chemo (70) 59%; RT (10) 8%; post-op (27) 23%; post-chemo (55) 47%; post-RT (54) 46%
Pancieri 2010 Brazil [22]	40	22/18	48 ± 16	6MWT; SCT	Hospital / consecutive	NSCLC (33) 82.5%; other LC (2) 5%; benign (5) 12.5%	NR	Pre-op LR
Kasymjanova 2009 Canada [25]	64	29/35	62.0 ± 10.8	6MWT	Outpt clinic / consecutive	NSCLC	III (8) 12%; IV (56) 87%	Pre-chemo
Mao 2007* Miller 2005 USA [26,27]	53	28/25	64 (range 45–81)	6MWT	Hospital/ sample part of larger prospective trial	NSCLC (39) 74%; SCLC (12) 23%; meso (1) 2%; lung met (1) 2%	NSCLC only: I – II (4) 8%; III-IV (41) 77%; recurrent (8) 15%	Pre- RT (17) 32%; pre-chemo-RT (36) 68%; prior chemo (33) 62%;
Saad 2007 Brazil [24]	36	20/16	Median 55.5 ± 13.4	6MWT	Hospital / consecutive	NSCLC (26) 72%; lung met (8) 22%; sarcoma (2) 6%	NR	Pre-op LR
Parsons 2003 Canada [39]	70	40/30	65 (range 29–83)	6MWT SCT	Hospital / convenience	NSCLC (55) 79%; pulmonary met (11) 16%; meso (2) 3%; benign (2) 3%	NR	Pre-op LR
Pierce 1994 Australia [23]	54	54/0	67 ± 7	6MWT	Hospital / consecutive	LC (including NSCLC)	NR	Pre-op LR
Holden 1992 USA [41]	16	13/3	68 ± 9.3	6MWT; SCT	Hospital	NSCLC (15) 94%; SCLC (1) 6%	I (10) 62%; II (3) 19%; III (2) 12%; IV (1) 6%	Pre-op LR
England 2012 UK [44]	41	21/20	64 ± 8	ISWT	Outpt clinic	NSCLC (26) 63%; meso (11) 27%; SCLC (4) 10%	Local (21) 51%; advanced (20); 49%	Post pall-chemo (26) 63%; post pall-RT (10) 24%; post-RT (1) 2%
Win 2006 UK [30]	125	81/44	68.8 ± 7.7	ISWT	Consecutive	NSCLC	NR	Pre-op LR
Win 2004 UK [64]	111	71/40	69 (range 42–85)	ISWT	Outpt clinic	NSCLC (107) 96%; miscellaneous (4) 4%	I-IIIA	Pre-op LR
Brunelli 2012 Italy [43]	282	218/64	68.0 ± 9.8	SCT	Tertiary referral centre / consecutive	NSCLC	I (118) 42%; other (164) 58%	Pre-op LR
Brunelli 2010 Italy [31]	109	83/26	66.6 ± 11.1	SCT	Tertiary referral centre / consecutive	NSCLC	NR	Pre-op LR
Brunelli 2008a Italy [34]	536	426/110	67 ± 9	SCT	Tertiary referral centre / consecutive	NSCLC	I (206) 38% ; > I (330) 62%	Pre-op LR (536) 100%; chemo (56) 10%
Brunelli 2008b Italy [33]	640	NR	66.7 ± 9.3	SCT	Tertiary referral centre / consecutive	NSCLC	NR	Pre-op LR (640) 100%; neoadjuvant chemo (100) 16%
Koegelenberg 2008 South Africa [29]	44	31/13	47.6 ± 12.5	SCT	Tertiary referral centre / consecutive	NSCLC (13) 29%; benign (31) 70%	NR	Pre-op LR
Nikolic 2008 Croatia [36]	101	82/19	61.1 ± 8.4	SCT	Hospital / consecutive	NSCLC	NR	Pre-op LR
Brunelli 2007 Italy [20]	200	NR	66.8 ± 9.1	SCT	Tertiary referral centre / consecutive	NSCLC	NR	Pre-op LR (200) 100%; neoadjuvant chemo (19) 9.5%
Toker 2007 Turkey [37]	150	127/23	59.3 ± 10.3 (gp 1) 60.7 ± 10.9 (gp 2)	SCT	University hospital / consecutive	NSCLC	NR	Pre-op LR
Brunelli 2005 Italy [42]	391	309/82	69.1 ± 8.3 (gp 1) 67.0 ± 9.0 (gp 2)	SCT	Tertiary referral centre / consecutive	NSCLC	NR	Pre-op LR
Brunelli 2004 Italy [35]	109	NR	75.2 ± 3.0	SCT	Tertiary referral centre / consecutive	NSCLC	I (23) 21% ; > I (86) 79%	Pre-op lobectomy
Brunelli 2003 Italy [21]	227	NR	66.4 ± 9.1 (gp 1) 66.8 ± 8.1 (gp 2)	SCT	Tertiary referral centre / consecutive	NSCLC	NR	Pre-op LR
Brunelli 2002 Italy [32]	160	128/32	66.2 ± 9.6	SCT	Tertiary referral centre / consecutive	NSCLC	NR	Pre-op LR
Pate 1996 USA [38]	12	10/2	63.6 ± 4.9	SCT	Three university hospitals / consecutive	NSCLC (7) 58%; NR (5) 42%	I (5) 42%; III (2) 17%; NR (5) 42%	Pre-op LR
**Physical activity**								
Maddocks 2012 UK [47]	84	54/30	66 ± 9	Acc	Outpt clinic	NSCLC (71) 84%; SCLC (8) 9%; meso (5) 6%	IIIB (43) 51%; IV (41) 49%	Palliative Rx
Grutsch 2011a, 2011b; Du-Quiton 2010 USA [48-50]	84	65/19	62 (range 40–94)	Acc	Hospital inpt (42) 50%; home (42) 50%	NSCLC	II (1) 1%; III (18) 21%; IV (65) 77%	Pre-chemo (84) 100%; prior Rx (31) 37%
Maddocks 2010 UK [46]	60	40/20	68 ± 9	Acc	Outpt clinic	NSCLC (53) 88%: meso (5) 8%; GI (2) 3%	Local (35) 58%; advanced (25) 42%	NR
Novoa 2011 Spain [51]	38	30/8	62.8 ± 10.1	Pedom	Output clinic / consecutive	NSCLC	NR	Pre-op LR
**Muscle strength**								
Trutschnigg 2008 Canada [28]	74	48/26	61.5 ± 13.1	HGD	Hospital and laboratory	NSCLC; GI	Advanced	NR
Brown 2005 UK [53]	53	30/23	Median 64 (range 43–81)	HGD CST	Palliative care centre x 2 and hospital	Gp1: healthy controls (15) 100% Gp2: cancer: NSCLC (29) 76%; SCLC (6) 16%; no histology LC (3) 8%	Locally advanced (20) 53%; IV (18) 47%	NR
Knols 2002 Switzerland [52]	40	27/13	49.4 ± 14.8	HHD+ pulley	Hospital inpt / convenience	NSCLC (7) 17.5%; other LC (3) 7.5%; haem (20) 50%; sarcoma (5) 12.5%; seminoma (3) 7.5%; other (2) 5%	I-IV	Chemo

Abbreviations: *6MWT*, six-minute walk test; *Acc*, accelerometer; *ca*, cancer; *chemo*, chemotherapy; *CST*, chair-stand test; *F*, female; *gp*, group; *gyn*, gynaecological; *haem,* haematological; *GI*, gastro-intestinal; *HHD*, hand-held dynamometry; *HGD*, hand-grip dynamometry; *HT*, hormone therapy; *ISWT*, incremental shuttle walk test; *inpt*, inpatient; *LC*, lung cancer; *LR*, lung resection; *M*, male; *meso*, mesothelioma; *met*, metastasis; *n*, number of participants; *NR*, not reported; *NSCLC*, non-small cell lung cancer; *OM*, outcome measure; *outpt*; outpatient; *pall*, palliative; *pedom*, pedometer; *PFS*, pulley-force sensor; *post-op*, post-operative; *pre-op*, pre-operative; *pt*, participant; *RT*, radiotherapy; *Rx*, treatment; *stair*, stair-climbing test; *SCLC*, small cell lung cancer; *SCT*, stair-climb test; *SD*, standard deviation; *wks*, weeks; *yr*, year published; *yrs*, years.

* data presented from most recent publication.

**Table 3:** Description of outcome measures used

**Author, yr** ***6MWT***	**OM procedure referenced**		**Encouragement standardised**		**Number of repeat Ax in testing session**		
Jones 2012 [40]	Yes [50]		NR		NR		
Pancieri 2010 [22]	No		‘encouraged walking’		NR		
Kasymjanova 2009 [25]	Yes [50,67]		NR		1 x practice 1 x actual (on different days)		
Mao 2007, Miller 2005 [26,27]	Yes [20]		NR		NR		
Saad 2007 [24]	Yes [50]		NR		NR		
Parsons 2003 [39]	Yes [34,68-72]		Yes		1 x practice, 1 x actual		
Pierce 1994 [23]	Yes [73]		NR		3 (best value used)		
Holden 1992 [41]	No		NR		2 (15-30 min interval)		
***ISWT***	**OM procedure referenced**		**Encouragement standardised**		**No of repeat Ax in testing session**		**Additional description**
England 2012 [44]	Yes [59]		NR		1 x practice, 1 x actual		Participants wore COSMED K4 b^2^ system (COSMED, Italy) to measure HR, VE and VO_2_ throughout test
Win 2006; 2004 [30,64]	Yes [59]		NR		1		HR and SpO_2_ monitored and recorded at 30 second intervals throughout test
***SCT***	**Equipment**	**Monitoring during test**	**Intensity**	**Outcome**	**Number of repeat Ax in testing session**	**Experience of assessors**	**Additional description**
Brunelli 2012, 2010, 2008a, 2008b, 2007, 2005, 2004, 2003, 2002 [20–21, 31, 32–35, 42–43,]	16 flights x 11 steps (0.155 m height)	Continuous verbal interaction to Ax SOB/ symptoms; HR; SpO_2_	Pace of pt choice, asked to climb max no steps and stop for exhaustion, limiting SOB, leg fatigue or chest pain	No steps and time taken, minimum value SpO_2_, exercise oxygen desaturation (= fall SpO_2_ < 90% or fall >4%)	1	Physician	Calculations from test:
							• Work = (step height in meters x steps per min x body weight in kg x 0.1635)[74]
							• VO_2_max (ml/min) = 5.8 x weight in kg + 151 + 10.1 x work) [74]
							• VO_2_max corrected for body surface area (ml/min^2^) = VO_2_max / max HR
Pancieri 2010 [22]	6 flights x 12 steps (16.9 cm height), 30° incline	HR, SpO_2_	Climb all steps in the shortest possible time with verbal encouragement between flights. Testing stopped for fatigue, limiting SOB, thoracic pain or exhaustion	Time taken	1		
Koegelenberg 2008 [29]	12 flights x 10 steps (3.48 m b/t floors)	HR, SpO_2_	Pt asked to climb ‘as fast and as high’ as they possibly could to a max elevation of 20 m	Altitude, time taken, speed of ascent	NR	NR	Stair climb considered completed if pt rested or more than 3 seconds or reached 20 m height Allowed to use rail only for balance
Nikolic 2008 [36]	92 steps (0.15 m height)	HR, SpO_2_ (measured every 20 steps)	Pace of pt choice, asked to climb max no steps and stop for exhaustion, limiting SOB, leg fatigue or chest pain	No steps, time taken	1	Physician	Pt instructed not to use hand-rail
Toker 2007 [37]	20 steps per flight (15 cm height)	HR, SpO_2_	Pt motivated to do their best and motivation	SpO_2_ (pre, post, change), HR (pre, post, change), time taken	2	Resident doctor	
Pate 1996 [38]	21 steps per flight (17.5 cm height)	HR, SpO_2_	Moderate pace of pts choice, encouraged to exercise to a symptom-limited max and complete the flight of stairs they were on if possible	No steps, time taken, altitude (m), reason for stopping	1	NR	Test considered completed as soon as patient stopped for any reason Pt instructed not to use hand-rail
Holden 1992 [41]	11 steps per flight (0.17 m height)	SpO_2_	Own pace	Altitude, time taken	1	Therapist	Calculations from test:
							• Work = step height x steps per min x weight in kg x 0.1635
							• VO_2_ ml/min = 5.8 x weight in kg + 151 + (10.1 x work)
***Physical activity***	**Equipment**		**Location of monitoring**	**Outcome**	**Duration of Ax**	**Position of sensor**	
Maddocks 2012 [47]	Uni-axial accelerometer ActivPAL^TM^ monitor (PAL Technologies Ltd., UK)	Home environment	Mean daily step count, number of sit-to-stand transitions, time in hrs spent sitting/lying, upright standing and upright stepping	7 days (6 full days of data)	Mid-third of anterior thigh of pts chosen leg		
Grutsch 2011a, 2011b; Du-Quiton 2010 [48-50]	Actigraph Piezoelectric Accelerometer (Ambulatory Monitoring Inc., AMI, USA)	Group 1 (inpt) and group 2 (home environment)	Mean duration daytime activity (no of vertical/horizontal accelerations per min)	3-7 days	Non-dominant wrist		
Novoa 2011 [51]	OMROM Walking Style Pro® pedometer	Home environment	Mean daily no total and aerobic steps; mean daily distance walked (km); mean daily time of aerobic activity (min)	Daytime only, daily Ax while waiting for surgery	Pedometer attached to waist band or belt		
Maddocks 2010 [46]	Uni-axial accelerometer ActivPAL^TM^ monitor (PAL Technologies Ltd., UK)	Home environment	Mean daily step count and estimate energy expenditure (METh); acceptability (non-compliance in hours); optimal duration of monitoring	7 days (6 full days of data)	Mid-third of anterior thigh of dominant leg		
***Physical activity***	**Equipment**		**Location of monitoring**	**Outcome**	**Duration of Ax**	**Position of sensor**	
Maddocks 2012 [47]	Uni-axial accelerometer ActivPAL^TM^ monitor (PAL Technologies Ltd., UK)	Home environment	Mean daily step count, number of sit-to-stand transitions, time in hrs spent sitting/lying, upright standing and upright stepping	7 days (6 full days of data)	Mid-third of anterior thigh of pts chosen leg		
Grutsch 2011a, 2011b; Du-Quiton 2010 [48-50]	Actigraph Piezoelectric Accelerometer (Ambulatory Monitoring Inc., AMI, USA)	Group 1 (inpt) and group 2 (home environment)	Mean duration daytime activity (no of vertical/horizontal accelerations per min)	3-7 days	Non-dominant wrist		
Novoa 2011 [51]	OMROM Walking Style Pro® pedometer	Home environment	Mean daily no total and aerobic steps; mean daily distance walked (km); mean daily time of aerobic activity (min)	Daytime only, daily Ax while waiting for surgery	Pedometer attached to waist band or belt		
Maddocks 2010 [46]	Uni-axial accelerometer ActivPAL^TM^ monitor (PAL Technologies Ltd., UK)	Home environment	Mean daily step count and estimate energy expenditure (METh); acceptability (non-compliance in hours); optimal duration of monitoring	7 days (6 full days of data)	Mid-third of anterior thigh of dominant leg		
***Muscle strength***	**Equipment**	**Muscle group movement**	**Participant position**	**No of assessors**	**Number of repeat Ax in testing session**	**Experience of assessors**	**Additional description**
Trutschnigg 2008 [28]	Jamar HGD (Sammons Preston, Bolingbrook), position 3 on handle	Grip	Sitting, feet on ground shoulder width apart, Elb 90 ° F, wrist 0°, arm on arm rest [75]	NR	1-2 x practice 2 x 3reps actual (mean value used)	NR	Dominant hand Patient instructed when to start and stop contraction with a 3 second contraction time No encouragement
Brown 2005 [53]	Square design chair with firm seat 43 cm height and arm rests	Sit to stand	Seated	NR	NR	NR	Pt asked to rise from seated position to fully upright position as fast as they could, if possible without using arm rests
Knols 2002 [52]	Mecmesin FB50K pull-gauge HHD, Mecmesin, England	Elb E, Knee E	1. Supine, Elb 90 °F, upper edge pull- attachment perpendicular to ulnar side of forearm distal to caput ulnae, non-elastic belt over ASIS stabilised pt on table	2 (random order) 30 min interval	6 x practice 3 x actual (60 second interval)	Physiotherapist	Dominant UL and LL Ax – identified by asking participant to throw a ball and kick a ball (preferred stance leg chosen) Figures also provided for both testing positions ‘Make’ test used Pt asked to increase force over 2 seconds and maintain for another 5 seconds
			2. sitting edge of table upright no back support, knee 90 °F, stabilise trunk by grasping table, lower edge pull-attachment perpendicular to ant surface tibia, distal to end med mall				

Abbreviations: *1RM*, one repetition maximum; *6MWT*, six-minute walk test; *ant*, anterior; *ASIS*, spinae iliacae ant superiorum; *ATS*, American Thoracic Society; *Ax*, assessment; *bt*, between; *cm*, centimeters; *dyn*, dynamometer; *E*, extension; *Elb*, elbow; *F*, flexion; *HHD*, hand held dynamometer; *HGD*, hand grip dynamometer; *HR*, heart rate; *hrs*, hours; *ISWT*, incremental shuttle walk test; *kg*, kilograms; *km*, kilometers; *L*, left; *LL*, lower limb; *m*, meters; *mal*, malleolus; *max*, maximum; *med*, medial; *METh*, metabolic equivalent hours; *min*, minutes; *ml*, millimetres; *NA*, not applicable; *no*, number; *NR*, not reported; *OM*, outcome measure; *pt*, participant; *R*, right; *reps*, repetitions; *SCT*, stair-climb test; *SOB*, shortness of breath; *SpO*_*2*_, oxygen saturation; *UL*, upper limb; *VE*, minute ventilation; *VO*_*2*_ oxygen uptake; *VO*_*2*_*max*; maximal oxygen consumption; *yr*, year published.

#### Outcome measures

Measurement properties evaluated were: intra-rater reliability (studies n=1); inter-rater reliability (n=1); measurement error (n=1); criterion-concurrent validity (n=2); criterion-predictive validity (n=20); construct validity (hypothesis testing) (n=11) and responsiveness (n=0) (Table 1; Table 4; Additional file 3).

**Table 4:** Criterion-concurrent validity, criterion-predictive validity and construct validity of outcome measures

**Author, yr**	**Type of validity and OM**	**Missing values**	**Comparator OM or predicted outcome**	**Validation results**
**Functional capacity**				
Jones 2012 [40]	Crit-pred 6MWT	Nil	All-cause mortality	Unadjusted HR p = 0.003; Compared to 6MWT <358.5 m adjusted HR = 0.61 (95% CI 0.34-1.07) if 6MWT 358.5-450 m; Compared to 6MWT <385.5 m adjusted HR = 0.48 (95% CI 0.24-0.93) if 6MWT >450 m
Pancieri 2010 [22]	Crit-pred 6MWT	NR	Predicted post-op 6MWT = pre-op 6MWT x (FS – resected FS) ÷ FS	r = 0.40, p<0.01
Kasymjanova 2009 [25]	Crit-pred 6MWT	19pts dropped out	Survival	Compared to 6MWT ≥ 400 m mortality HR = 0.44 (95% CI 0.23-0.83) if 6MWT <400 m, p = 0.001
Mao 2007* Miller 2005 [26,27]	Crit-pred; construct 6MWT	3pts not complete Ax	1. Development of RP	1. ROC area under curve = 0.41, p = 0.4
			2. FEV_1_	2. r = 0.53, p<0.001
			3. FVC	3. r = 0.44, p = 0.001
			4. DLCO	4. r = 0.48, p<0.001
Saad 2007 [24]	Crit-pred 6MWT	9pts died; 30pts not complete Ax (rural)	Predictors of improvement in pre-op to 180 days-post-op:	1. GEE = 0.001, SE = 0.000, p = 0.003
			1. SF-36 PF	2. GEE = 0.001, SE = 0.000, p = 0.000
			2. SF-36 PR	3. GEE = 0.001, SE = 0.000, p = 0.031
			3. SF-36 GH	
Parsons 2003 [39]	Crit-pred 6MWT	29pts	LOS out of hospital < 30 days post-op	Not significant
Pierce 1994 [23]	Crit-pred 6MWT	NR	Post-op:	1. p<0.05
			1. respiratory failure	2. p>0.05
			2. surgical POC	3. p>0.05
			3. respiratory POC	4. p>0.05
			4. cardiac POC	5. p>0.05
			5. all POC	
Holden 1992 [41]	Crit-pred 6MWT	3pts not complete Ax	Survival > 90 days post-op	6MWT diff b/t groups with/without survival p<0.05;
				6MWT > 1000feet (305 m) pre-op sensitivity 100%, positive predictive value 85%, negative predictive value 100% for survival
England 2012 [44]	Construct ISWT	Nil	1. P max monitor (insp mm strength)	1. r = 0.42, p = 0.01
			2. dynamometry (peripheral mm power)	2. r = 0.39, p = 0.01
			3. spirometry (% predicted FEV_1_)	3. r = 0.22, p = 0.17
			4. spirometry (% predicted FVC)	4. r = 0.21, p = 0.2
			5. CRDQ (mastery)	5. r = 0.21, p = 0.18
Win 2006 [30]	Crit-pred; crit-concurrent; construct ISWT	Nil	1. CPET (VO_2peak_)	1. r = 0.67, p<0.001
			2. CPET (VO_2peak_% predicted)	2. r = 0.30
			3. spirometry (FEV_1_)	3. r = 0.5
			4. 12 month survival	4. ROC area = 0.7, p = 0.003
Win 2004 [64]	Crit-pred, construct ISWT	8pts	1. poor surgical outcome (post-op death, MI, heart failure, resp failure, septicaemia, pneumonia, cardiac arrthymia)	1. p = 0.6 between poor and sufficient outcome groups
			2. FEV_1_	2. r = 0.46, not significant
Brunelli 2012 [43]	Crit-pred SCT	14	Median survival and 5-year survival	Altitude >18 m independent predictor: HR = 0.5, p = 0.003
Brunelli 2010 [31]	Construct SCT	Nil	SCT (VO_2max_)	Altitude: correlation coefficient = 0.7, p<0.0001
				Speed of ascent: correlation coefficient = 0.47, p = 0.005
Pancieri 2010 [22]	Crit-pred SCT	NR	Predicted post-op SCT = pre-op SCT x (FS – resected FS) ÷ FS	r = 0.66, p<0.001
Brunelli 2008a [34]	Crit-pred SCT	Nil	POC < 30 days post-op	Pre-op altitude: coefficient = −0.05, OR = 0.95 (95% CI 0.91-0.99), SE = 0.02, p = 0.045
				O_2_desat >4%: coefficient = 0.56, OR = 1.8 (95% CI 1–3), SE = 0.3, p = 0.05
Brunelli 2008b [33]	Crit-pred SCT	Nil	1. POC < 30 days post-op	1. altitude: coefficient = 0.34, SE = 0.2, OR = 1.4 (95% CI 1.02-1.95), p = 0.04
			2. Death < 30 days post-op	2. altitude: coefficient = 0.91, SE = 0.4, OR = 2.5 (95% CI 1.1-5.5), p = 0.02
			3. Post-op hospital costs	3. altitude: coefficient = 2160.2, SE = 573, p<0.001
Koegelenberg 2008 [29]	Crit-concurrent SCT	Nil	CPET (VO_2max_)	Altitude r^2^ = 0.06, Speed of ascent r^2^ = 0.77 (lung cancer only)
Nikolic 2008 [36]	Crit-pred SCT	Nil	POC < 30 days post-op	Best independent predictor = SpO_2_ after 40 steps and SCT duration for lobectomy group (60% sensitivity, 75% specificity cut off value 1.09) positive LR = 2.4 (95% CI 1.71-3.38), negative LR = 0.53 (95% CI 0.38-0.76)
Brunelli 2007 [20]	Constuct SCT	53pts at 3 months	Post-op SCT (VO_2_peak)	Pre-op SCT VO_2_peak directly associated with post-op SCT: regression analysis lobectomy F = 3.58, p<0.01; pneumonectomy F = 3.53, p<0.01
Toker 2007 [37]	Crit-pred SCT	Nil	POC (cardiac or pulmonary)	SpO_2_ pre-SCT: OR = 0.74 (95% CI 0.58-1.00), p = 0.001
				Change SpO_2_ pre to post-SCT: OR = 1.59 (95% CI 1.21-2.10), p = 0.018
Brunelli 2005 [42]	Crit-pred SCT	13pts	1. POC < 30 days post-op	Inability to perform pre-op SCT:
				1. p = 0.7
			2. Death < 30 days post-op	2. OR = 0.20 (95% CI 0.06-0.62), p = 0.005
Brunelli 2004 [35]	Crit-pred SCT	18pts	POC < 30 days post-op	Lower altitude pre-op independent predictor: coefficient = −0.18, p = 0.0015
Brunelli 2003 [21]	Crit-pred SCT	Nil	O_2_ desat during post-op SCT	O_2_ desat during pre-op SCT independent variable: regression coefficient = 0.22, p = 0.0004
Parsons 2003 [39]	Crit-pred SCT	29pts	LOS out of hospital < 30 days post-op	1. longer LOS correlated with speed of ascent r = 0.34, p≤0.02
				2. workload achieved predicted LOS out of hospital r^2^ = 0.130
Brunelli 2002 [32]	Crit-pred SCT	Nil	POC < 30 days post-op	Altitude independent variable: p = 0.003
Pate 1996 [38]	Crit-pred SCT	Nil	POC < 30 days post-op	Significant difference in pre-op SCT between pt who did and did not develop POC
Holden 1982 [41]	Crit-pred SCT	3pts	Survival > 90 days post-op	SCT diff b/t groups with/without survival p<0.05
				SCT > 44steps pre-op positive predictive value 91%, negative predictive value 80% for survival
**Physical activity**				
Maddocks 2012 [47]	Construct Acc	Nil	ECOG PS	Statistically significant difference in mean daily step count, time spent sitting/lying, upright, standing or stepping between PS 0, 1 and 2 p<0.05 but not mean daily sit-to-stand transitions
Grutsch 2011a, 2011b; Du-Quiton 2010 [48-50]	Construct Acc	16pts Acc, 16pts questionnaires	1. HADS	1. Outpt: depression and activity r = −0.41, p = 0.04
			2. Ferrans and Power QLI Cancer Version III	2. Daytime activity and QLI domains of health/functioning r = 0.51, p<0.01; social/economic r = 0.38, p = 0.048; psychological/spiritual r = 0.45, p = 0.02; family r = 0.45, p = 0.02; overall QLI r = 0.57, p<0.01
			3. EORTC	3. Inpt: daytime activity and loss of appetite r = −0.41, p = 0.005
			4. PSQI	4. Outpt: lower sleep medication use and activity r = −0.58, p<0.01
Novoa 2011 [51]	Construct Pedom	13 pts – unable to perform exercise test	CPET (VO_2max_)	Mean daily total steps r = 0.4
				Mean daily aerobic steps r = 0.16
				Mean daily time of aerobic capacity r = 0.11
				Mean daily distance walked r = 0.44
Maddocks 2010 [46]	Construct Acc	2 pts withdrawn	Estimated EE (stepping and non-stepping) measured from acc	Non-stepping EE and daily step count r = −0.91, p<0.01
**Muscle strength**				
Brown 2005 [53]	Construct CST	Nil	1. KPS	1. r^2^ = 0.565, p < 0.001 (ca group)
			2. FACIT-fatigue	2. with incr fatigue, lower CST p<0.01 (ca group)

Abbreviations: *6MWT*, six minute walk test; *95% CI*, 95% confidence intervals; *acc*, accelerometery; *Ax*, assessment; *b/t*, between; *ca*, cancer; *CPET*, cardio-pulmonary exercise testing; *CRDQ*, Chronic Respiratory Disease Questionnaire; *crit*, criterion; *CST*, chair-stand test; *desat*, desaturation; *diff*, difference; *DLCO*, diffused capacity for carbon monoxide; *ECOG*, Eastern Cooperative Oncology Group; *EE*, energy expenditure; *EORTC*, European Organisation for Research and Treatment of Cancer quality of life questionnaire; *FACIT-fatigue*, Functional Assessment of Chronic Illness Therapy- Fatigue scale; *FEV*_*1*_, force expired volume in one second; *FS*, lung functioning segments; *FVC*, forced vital capacity; *GEE*, generalized estimation equations; *HADS*, Hospital Anxiety and Depression Scale; *HR*, hazard ratio; *insp*, inspiratory; *inpt*, inpatient; *ISWT*, incremental-shuttle walk test; *KPS*, Karnofsky performance status; *LOS*, length of stay; *LR*, likelihood ratio; *m*, meters; *max*, maximum; *MI*, myocardial infarction; *mm*, muscle; *NR*, not reported; *OM*, outcome measure; *OR*, odds ratio; *outpt*, outpatient; *P*, pressure; *pedom*, pedometer; *POC*, post-operative complication; *post-op*, post-operative; *pre-op*, pre-operative; *pred*, predictive; *PS*, performance status; *pt*, participant; *PSQI*, Pittsburgh Sleep Quality Index; *QLI*, Quality of Life Index; *r*, correlation coefficient; *ROC*, received operating characteristic curve; *SCT*, stair-climb test; *SE*, standard error; *SF-36*, Short Form 36 physical functioning/physical role/general health domain; *resp*, respiratory; *RP*, radiation pneumonitis; *Rx*, treatment; *SpO*_*2*_, oxygen saturation; *VO*_*2max*_, maximum oxygen consumption; *VO*_*2peak*_, peak oxygen consumption; *yr*, year published.

* results presented from most recent publication.

#### Risk of bias of studies

Risk of bias was assessed by independent reviews (CG, CO) achieving a percentage agreement of 87%, Kappa=0.80 [16]. Consensus was achieved on 100% of occasions that reviewers disagreed. Overall studies evaluating validity scored ‘excellent’ or ‘good’ on 12/29 occasions. No studies evaluating reliability scored ‘excellent’ or ‘good’ (Table 5). The worst performing area for validity studies was design requirements (lack of a priori hypotheses formed) and for reliability studies was design requirements (small sample size).

**Table 5:** Methodological quality of included studies - part two

**Author, yr**	**Reliability**	**Measurement error**	**Hypothesis testing**	**Criterion validity**	**Responsiveness**
**Functional capacity**					
Brunelli 2012 [43]	x	x	x	Fair	x
England 2012 [44]	x	x	Fair	x	x
Jones 2012 [40]	x	x	x	Excellent	x
Brunelli 2010 [31]	x	x	Excellent	x	x
Pancieri 2010 [22]	x	x	x	Fair	x
Kasymjanova 2009 [25]	x	x	x	Fair	x
Brunelli 2008a [34]	x	x	x	Excellent	x
Brunelli 2008b [33]	x	x	x	Excellent	x
Koegelenberg 2008 [29]	x	x	x	Fair	x
Nikolic 2008 [36]	x	x	x	Poor	x
Brunelli 2007 [20]	x	x	Fair	x	x
Mao 2007 [26]	x	x	Fair	Good	x
Saad 2007 [24]	x	x	x	Poor	x
Toker 2007 [37]	x	x	x	Excellent	x
Win 2006 [30]	x	x	Fair	Good	x
Brunelli 2005 [42]	x	x	x	Excellent	x
Brunelli 2004 [35]	x	x	x	Good	x
Win 2004 [64]	x	x	Poor	Poor	x
Brunelli 2003 [21]	x	x	x	Excellent	x
Parsons 2003 [39]	x	x	x	Good	x
Brunelli 2002 [32]	x	x	x	Excellent	x
Pate 1996 [38]	x	x	x	Poor	x
Pierce 1994 [23]	x	x	x	Poor	x
Holden 1992 [41]	x	x	x	Poor	x
**Physical activity**					
Maddocks 2012 [47]	x	x	Poor	x	x
Novoa 2011 [51]	x	x	Poor	x	x
Grutsch 2011a, 2011b; Du-Quiton 2010 [48-50]	x	x	Fair	x	x
Maddocks 2010 [46]	x	x	Fair	x	x
**Muscle strength**				x	x
Trutschnigg 2008 [28]	Poor (intra-r)	x	x	x	x
Brown 2005 [53]	x	x	Fair	x	x
Knols 2002 [52]	Fair (inter-r)	Fair	x	x	x

Abbreviations: *inter-r*, inter-rater reliability; *intra-r*, intra-rater reliability; *yr*, year published.

x = not assessed.

#### Study results

Study results are summarised in Table 1 and the sections below. The stair-climbing test, six-minute walking test (6MWT) and incremental-shuttle walk test (ISWT) performed the best out of the 13 tests reviewed, primarily due to lack of studies investigating measurement properties of the other 10 tests (Table 1).

##### Functional capacity

The 6MWT, twelve-minute walking test (12MWT), ISWT, endurance-shuttle walking test (ESWT) and stair-climbing test are field tests reflecting functional capacity. No studies investigated inter or intra-rater reliability, measurement error or responsiveness of these tests in participants with NSCLC.

The criterion-concurrent validity of the ISWT and stair-climbing test against the gold standard cardio-pulmonary exercise test (CPET) was reported by three studies (Table 4) [29-31]. The ISWT was validated against CPET (VO_2_peak) with strong correlation (r=0.67) [30]. The stair-climbing test (ascent speed) was validated against CPET (maximum oxygen consumption VO_2_max) with strong correlation (r^2^=0.77) [29].

The criterion-predictive validity of the 6MWT, ISWT and stair-climbing test were reported and these instruments were shown to predict post-operative outcomes (studies n=12) [20-24,32-38], post-operative length of hospital stay (n=1) [39] and survival (n=8) (Table 4) [23,25,30,33,40-43]: Pre-operative stair-climbing test was a predictor for post-operative complications when using variables: test duration [36], oxygen saturation [34,36,37] or altitude [32-35,38]. Pre-operative 6MWT was a predictor for post-operative respiratory failure (p<0.05) [23]. Pre-operative stair-climbing test was a predictor for post-operative length of stay (r=0.34) [39] and hospital cost (coefficient=2160.2) [33]; and 6MWT was a predictor for post-operative health related quality of life (HRQoL) physical domains (GEE=0.001) [24]. The 6MWT was shown in two papers to predict survival in advanced NSCLC (hazard ratios=0.44 [25] and 0.48 [40]). With every 50 m improvement in 6MWT, survival improved by 13% [40] and patients walking ≥ 400 m pre-chemotherapy had greater survival time [25]. In the post-operative population survival was predicted by pre-operative ISWT (area under the ROC curve=0.7) [30]; stair-climbing test (steps climbed) (p<0.05) [41]; stair-climbing test (altitude) (coefficient=0.91 [33]; hazard ratio=0.5 [43]) and inability to perform stair-climbing test (odds ratio=0.2) [42]. A pre-operative stair-climbing test result of >44steps predicted post-operative survival at 30 days (positive predictive value=91%, negative predictive value=80%) [41].

Three studies reported on the construct validity of the 6MWT and ISWT: The 6MWT was validated against respiratory function tests (forced expired volume in one-second) with strong correlation (r=0.53) [26]. The ISWT was validated with moderate correlation against inspiratory muscle strength (r=0.42) [44] and isokinetic muscle dynamometry (r=0.39) (Table 4) [44].

##### Physical activity

No studies validated accelerometers or pedometers against the gold standard measure of physical activity (direct calorimetry) [45] or investigated reliability, measurement error or responsiveness. Four studies investigated construct validity (Table 4): The ActivPAL™ accelerometer (step count) was validated against ActivPAL™ (estimated energy expenditure) with strong correlation (r=−0.91) [46] and Eastern Cooperative Oncology Group (ECOG) Performance-Scale (p<0.05) [47]. The Actigraph (accelerations/minute) was validated with medium correlation against the Hospital Anxiety and Depression Scale (depression) (r=−0.41) [48], the Ferrans and Power Quality of Life Index Cancer-Version III (HRQoL) (r=0.38-0.57) [49], the European Organisation for Research and Treatment of Cancer quality of life questionnaire (loss of appetite) (r=−0.41) [49]; and with strong correlation against the Pittsburgh Sleep Quality Index (sleep medication use) (r=−0.58) [50]. The OMROM Walking Style Pro® pedometer (distance walked) was validated against CPET (VO_2_max) with moderate correlation (r=0.4) [51].

##### Muscle strength

Only two studies investigated muscle strength test reliability (Table 1; Additional file 3): The inter-rater reliability of the MFB50K pulley-gauge hand-held dynamometer (HHD) (elbow/knee extension) was very good (ICC=0.90, 0.96 respectively), however measurement error between examiners was large (SEM=10.6, 19.8 respectively), as was the smallest detectable difference (SDD=29.4, 54.8 respectively) (Additional file 4) [52]. The Jamar hand-grip dynamometer (HGD) (grip-strength) intra-rater reliability percent coefficient of variation was 6.3, which was better than that demonstrated for HGD with Biodex attachment (%CV16.7) (Table 1; Additional file 3) [28].

No tests measuring muscle strength were validated against the gold standard measure (isokinetic dynamometry). Construct validity was reported for the chair-stand test with a moderate correlation against Karnofsky Performance Status (r^2^=0.56) (Table 4) [53].

## Discussion

This review focused on three commonly assessed outcomes (functional capacity, physical activity and muscle strength) used in the NSCLC literature [3]. Tests used to evaluate the effectiveness of exercise in patients with NSCLC must be reliable and responsive to change in the outcome of interest, regardless of the cancer stage of participants and therefore understanding how different NSCLC stages respond to the outcome measures is vital. Standardised measures allow generalizability of study results across trials, which is important in NSCLC, given the poor participant consent/retention rate [54] and mortality rate. The gold standard measurement of functional capacity, physical activity and muscle strength require laboratory tests which have significant limitations for use in exercise-based NSCLC research trials. CPET (functional capacity) [7], direct calorimetry (physical activity) and isokinetic dynamometry (muscle strength) require expensive equipment, advanced monitoring and experienced technicians. Whilst limited studies have reported CPET to be safe and feasible in NSCLC [55], field tests which be performed reliably in clinical settings may reduce research costs, participant burden and drop-out rates. This review demonstrated the use of 13 different field tests and, although a number of studies investigated the validity of outcome measures in NSCLC, only two studies investigated reliability, with no study investigating test responsiveness. Further studies are needed to establish measurement properties of standardised field tests for individuals with NSCLC to allow the most appropriate choice of test when designing research trials.

Functional capacity was the most common outcome of interest in this review, with the 6MWT most commonly used. Search 1 retrieved 38 studies utilising the 6MWT in NSCLC and Search 2 retrieved seven studies investigating 6MWT measurement properties. Only 51% (n=17/33) of studies published after 2002, using the 6MWT in Search 1, referenced the American Thoracic Society guidelines in their methodology [56]. Three studies referenced the guidelines but stated they performed only one 6MWT during a testing session. Two tests have been shown to enhance reliability in other populations, with reports demonstrating the second 6MWT increases by 9-15 m [56,57]. The encouragement used in the 6MWT in part one studies was variable. No studies identified in part two of this review analysed the reliability of the 6MWT. Similarly, in Search 1, 14 studies used the 6MWT to evaluate the benefit of exercise intervention over time, however no studies in Search 2 investigated the responsiveness of the 6MWT in any stage of NSCLC. In comparison, there has been a substantial amount of work regarding the criterion-predictive validity of the 6MWT in patients with NSCLC. Results demonstrated the 6MWT was predictive for post-operative complications, HRQoL and survival. The 6MWT has not been validated against CPET in NSCLC, however it has been validated against CPET in populations with cardiorespiratory disease with moderate correlations (r=0.51–0.93) [58-61]. Given the frequent use of the 6MWT, establishing reliability, measurement error, minimal clinically important difference, responsiveness and validating the 6MWT against CPET in NSCLC should be a priority.

In Search 1 the ISWT was used in six studies involving participants with NSCLC and twice this was to evaluate the benefit of exercise [62,63]. Only fifty percent of the studies described how the participant was monitored during the test [30,44,64], however all studies referenced their procedure, most (n=5/6, 83%) referencing the original protocol when the test was created [65]. The ISWT was only performed once during the testing session across all studies excluding one. Given no studies in Search 2 investigated the reliability of this test, similar to the case with the 6MWT, further research needs to investigate the best method for completing it in NSCLC to determine if a familiarisation effect is present.

The 12MWT and the ESWT have been infrequently used in studies of NSCLC and neither test was investigated regarding its measurement properties in NSCLC. Currently the alternative 6MWT and ISWT appear to be better choices of tests until further research is completed.

Search 1 identified 21 studies utilising the stair-climbing test in NSCLC, all in pre-lung resection candidates. No studies have used the stair-climbing test to evaluate exercise intervention. Currently there is no gold standard method to perform the stair-climbing test. Published studies used variable instructions, encouragement, monitoring and experience of assessors. Some authors reported the number of steps/altitude whilst others reported test duration. Results of Search 2 consistently demonstrated the stair-climbing test to be valuable in the pre-operative evaluation of lung resection candidates, with the stair-climbing test providing prediction validity with regard to post-operative complications, length of stay, mortality and hospital cost. The stair-climbing test has also been validated against the gold standard (CPET). No studies evaluated reliability; measurement error or responsiveness in NSCLC and therefore it is currently not known if this is a suitable test to evaluate exercise interventions, especially in post-operative and chemo-radiation cohorts.

Search 1 demonstrated that physical activity has been measured in participants with NSCLC using accelerometers and pedometers. Search 2 showed that accelerometers and pedometers have not been validated against the gold standard measure (direct calorimetry) in NSCLC. Direct calorimetry has limitations and accelerometers are commonly the preferred method to measure physical activity [66,67]. However, accelerometers and pedometers are limited in that they rely on participant compliance. In the NSCLC literature, few studies are conducted measuring physical activity levels and even fewer studies have investigated the measurement properties associated with tests.

Muscle strength was measured using five different tests by 17 studies in sSarch 1. Search 2 retrieved three studies evaluating measurement properties of only three of the five instruments. All three studies were conducted with mixed cancer cohorts and the methodological quality of each study was ‘poor’ or ‘fair’: therefore results need to be interpreted with caution. Hand dynamometry was the most commonly used instrument to assess muscle strength in part one studies. Two hand-dynamometry devices were tested for reliability however results were not strong enough to recommend use of a particular device. Whilst both HHD and HGD have been shown to be reliable and valid in many patient populations, further research needs to be performed in NSCLC [68-70]. Manual muscle testing is often considered to be qualitative and frequently performed in profoundly weak populations such as those with critical illness [71,72]. Four studies in Search 1 used MMT to measure upper-body strength on repeated occasions however the measurement properties have not been established. This review demonstrated that HHD, HGD, MMT, one-repetition maximum and the chair-stand test have been used in NSCLC, however there is currently insufficient research to support the use of one measure over another.

### Limitations

To minimise risk of selection bias two independent reviewers were utilised. In Search 2 articles were excluded if cancer type was unconfirmed. There is a risk of publication bias, where studies which have found poor measurement properties have not been published. Given that registration of studies evaluating measurement properties is not standard practice, the extent of this is unknown [8].

The COSMIN checklist was not completed in its entirety and may have also under-estimated methodological quality because the rating of each item was determined using the lowest score rather than the average or highest score.

Due to the small number of studies evaluating measurement properties of the included outcome measures in cohorts with only NSCLC participants, this review included studies with mixed cancer types (providing at least five participants had NSCLC). Different cancer types are associated with heterogeneous symptom profiles (for example dyspnoea and pain), gas exchange and exercise capacity. Therefore findings from the studies with mixed cancer types must be interpreted with caution when extrapolated for use in NSCLC. Additionally there was heterogeneity with regards to the participants in the included studies (particularly age and treatment exposure) (Table 2). This may explain, in part, the variance in data obtained and large standard deviations reported by individuals studies (Additional file 4) because age, comorbidities (such as COPD) and treatment (such as chemotherapy) directly impact exercise capacity and performance as well as the disease of NSCLC.

## Conclusion

Measurements of functional capacity, physical activity and muscle strength are commonly used as outcomes for individuals with NSCLC participating in exercise trials. The 6MWT, 12MWT, ISWT, ESWT and stair-climbing test have been used to assess functional capacity in NSCLC. Only two tests (ISWT and stair-climb test) were validated against CPET, the gold standard measure of functional capacity. Physical activity has been measured using accelerometers and pedometers: there was some evidence for construct validity but neither had been validated against the gold standard or tested for reliability. Muscle strength has been measured using HHD, HGD, manual muscle test, 1RM and the chair-stand test. Only two strength measures were tested for their reliability in NSCLC, and there was insufficient evidence to support the use of one strength measure over another. Responsiveness and minimal important clinical difference was not established for any of the 13 tests. Currently there is an important gap in the literature regarding the measurement properties of commonly used tests in NSCLC and further research needs to be conducted in this area to improve the clinical use and applicability of these tests in patients with NSCLC.

## Competing interest

The authors declare that they have no competing interests.

## Authors’ contribution

CG participated in the design of the protocol, contributed to establishment of search terms, performed database searching, reviewed articles for inclusion from Search 1 and 2 (as first independent reviewer), performed quality appraisal (as first independent quality assessor) and drafted the manuscript. CMcD participated in the design of the protocol, contributed to the background literature search and manuscript preparation. SP reviewed articles for inclusion from Search 1 and 2 (as second independent reviewer), cross checked extracted data and contributed to manuscript preparation. CO contributed to establishment of search terms, development of data extraction forms, performed quality appraisal (as second independent quality assessor) and contributed to manuscript preparation. LD participated in the design of the protocol, background literature search, contributed to the development of the search strategy, reviewed articles for inclusion from Search 1 and 2 (as third independent reviewer) and contributed to the manuscript preparation. All authors read and approved the final manuscript.

## Pre-publication history

The pre-publication history for this paper can be accessed here:

http://www.biomedcentral.com/1471-2407/13/135/prepub

## Supplementary Material

Additional file 1 **Flow diagram of outcome measures selection process – Search 1 **[11]**.** Abbreviations: Ax, assessment; CINAHL, Cumulative Index to Nursing and Allied Health Literature; DARE, Database of Abstracts and Reviews of Effects; EMBASE, the Excerpta Medica Database; FT, full text; n, number; NSCLC, non-small cell lung cancer; OM, outcome measure; PEDRO, Physiotherapy Evidence Database; PROM, patient reported outcome measure. Click here for file

Additional file 2 **Search strategy – Search 2 **[17]**.** Abbreviations: CINAHL, Cumulative Index to Nursing and Allied Health Literature; EMBASE, the Excerpta Medica Database; MESH, Medical Subject Heading Indexing. Click here for file

Additional file 3 **Interpretability.** Abbreviations: 6MWT, six minute-walk test; acc, accelerations; chemo, chemotherapy; CST, chair-stand test; E1, examiner one; E2, examiner two; Elb, elbow; E, extension; ECOG, Eastern Cooperative Oncology Group; ft, feet; gp, group; HGS, hand grip strength; hrs, hours; inpt, inpatients; IQR, inter-quartile range; ISWT, incremental-shuttle walk test; kg, kilogram; lbs, pounds; m, meters; MIC, minimal important change; min, minutes; ml, millilitres; N, newtons; outpt, outpatient; O_2_desat, oxygen desaturation; POC, post-operative complication; post-op, post-operative; pre-op, pre-operative; PS, performance status; RT, radiotherapy; s, seconds; SCT, stair-climb test; SD, standard deviation; SDD, smallest detectable difference; VO_2_peak, peak oxygen consumption; yr, year published.* results presented from most recent publication. Click here for file

Additional file 4 **Inter-rater reliability, intra-rater reliability and measurement error associated with outcome measures.** Abbreviations: 95% CI, 95% confidence intervals;%CV, percent coefficient of variation; b/t, between; E, extension; Elb, elbow; HGD, hand-grip dynamometry; HHD, hand-held dynamometry; ICC, intraclass correlation coefficient; mean diff, mean difference for repeated measures; min, minutes, OM, outcome measure; NR, not reported; SEM, standard error of measurement. Click here for file
