# Supplementary figures and images for: Functional capacity, physical activity and muscle strength assessment of individuals with non-small cell lung cancer: a systematic review of instruments and their measurement properties
Source: BMC Cancer. 2013 Mar 20;13:135. doi: 10.1186/1471-2407-13-135 (PMC3623892; doi:10.1186/1471-2407-13-135)

**Appendix 1: Flow diagram of outcome measures selection process – Search 1 [11]**

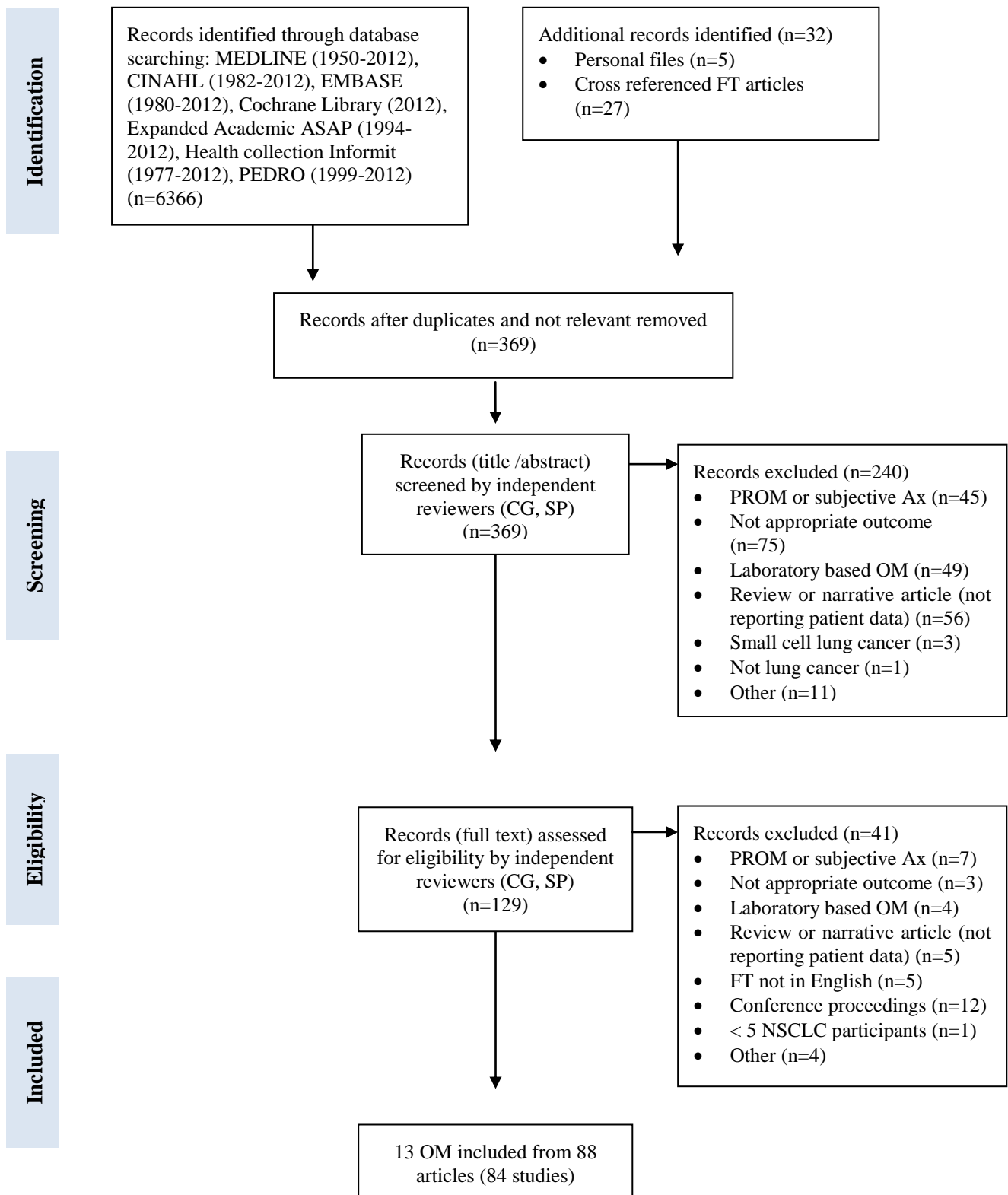

Supplement: Additional file 1 — Flow diagram of outcome measures selection process – Search 1 [11]. Abbreviations: Ax, assessment; CINAHL, Cumulative Index to Nursing and Allied Health Literature; DARE, Database of Abstracts and Reviews of Effects; EMBASE, the Excerpta Medica Database; FT, full text; n, number; NSCLC, non-small cell lung cancer; OM, outcome measure; PEDRO, Physiotherapy Evidence Database; PROM, patient reported outcome measure. [file 1471-2407-13-135-S1.pdf]
