# Supplementary material for: Functional capacity, physical activity and muscle strength assessment of individuals with non-small cell lung cancer: a systematic review of instruments and their measurement properties
Source: BMC Cancer. 2013 Mar 20;13:135. doi: 10.1186/1471-2407-13-135 (PMC3623892; doi:10.1186/1471-2407-13-135)
Supplement: Additional file 2 — Search strategy – Search 2 [17]. Abbreviations: CINAHL, Cumulative Index to Nursing and Allied Health Literature; EMBASE, the Excerpta Medica Database; MESH, Medical Subject Heading Indexing. [file 1471-2407-13-135-S2.docx]

Appendix 2: Search strategy – Search 2 [[17](#_ENREF_17)]

| **Database** | **Search fields** | **Search terms** |
| --- | --- | --- |
| CINAHL  EMBASE  Medline  Cochrane Library | Title, abstract, keywords | 1. “MH=(“psychometrics”) OR MH=(“observer variation”) OR MH=(“reproducibility of results”) OR MH=(“discriminant analysis”) OR TS=(clin?metr*) OR TS=(valid*) OR TS=(person?metric) OR TS=(reliab*) OR TS=(unidimensional*) OR TS=(discriminability) OR TS=(appropriateness) OR TS=(precision) OR TS=(interpretability) OR TS=(acceptability) OR TS=(practicability) OR TS=("floor effect") OR TS=("ceiling effect") OR TS=("standardised response mean") OR TS=(SRM) OR TS=("Guyatt's responsiveness statistic") OR TS=("effect size") OR TS=(“Validation Studies”) OR TS=(“Comparative Study”) OR TS=(“psychometr*”) OR TS=(“clinimetr*”) OR TS=(“observer variation”) OR TS=(reproducib*) OR TS=(“unreliab*”) OR TS=(coefficient) OR TS=(homogeneity) OR TS=(homogeneous) OR TS=(“internal consistency”) OR TS=(“cronbach* alpha*”) OR TS=(“item correlation*”) OR TS=(“item selection*”) OR TS=(“item reduction*”) OR TS=(agreement) OR TS=imprecision OR TS=(“precise values”) OR TS=(“test retest”) OR TS=(“test-retest”) OR TS=(“reliab* test”) OR TS=(“reliab* retest”) OR TS=stability OR TS=interrater OR TS=inter-rater OR TS=intrarater OR TS=intra-rater OR TS=intertester OR TS=inter-tester OR TS=intratester OR TS=intra-tester OR TS=interobserver OR TS=inter-observer OR TS=intraobserver OR TS=intraobserver OR TS=intertechnician OR TS=inter-technician OR TS=intratechnician OR TS=intra-technician OR TS=interexaminer OR TS=inter-examiner OR TS=intraexaminer OR TS=intra-examiner OR TS=interassay OR TS=inter-assay OR TS=intraassay OR TS=intra-assay OR TS=interindividual OR TS=inter-individual OR TS=intraindividual OR TS=intra-individual OR TS=interparticipant OR TS=inter-participant OR TS=intraparticipant OR TS=intra-participant OR TS=kappa OR TS=kappa’s OR TS=kappas OR TS=(repeatab*) OR TS=(“replicab* measure*”) OR TS=(“replicab* findings”) OR TS=(“replicab* result*”) OR TS=(“replicab* test*”) OR TS=(“repeated measure*”) OR TS=(“repeated findings”) OR TS=(“repeated result*”) OR TS=(“repeated test*”) OR TS=generaliza* OR TS=generalisa* OR TS=concordance OR TS=(“intraclass correlation*”) OR TS=discriminative OR TS=(“known group”) OR TS=(“factor analysis”) OR TS=(“factor analyses”) OR TS=dimension* OR TS=subscale* OR TS=(“multitrait scaling analys*”) OR TS=(“item discriminant”) OR TS=(“interscale correlation*”) OR TS=error* OR TS=(“individual variability”) OR TS=(“variability analys*”) OR TS=(“variability values”) OR TS=(“uncertainty measurement) OR TS=(“uncertainty measuring) OR TS=(“standard error of measurement”) OR TS=(sensitiv*) OR TS=responsive* OR TS=("minimal* detectable change") OR TS=(MDC) OR TS=(“minimal* clinical* important* difference”) OR TS=(“minimal* clinical* significant difference”) OR TS=(“minimal* clinical* detectable* difference”) OR TS=(“minimal* clinical* important* change”) OR TS=(“minimal* clinical* significant change”) OR TS=(“minimal* clinical* detectable* change”) OR TS=(“clinical* important* difference”) OR TS=(“clinical* significant difference”) OR TS=(“clinical* detectable* difference”) OR TS=(“clinical* important* change”) OR TS=(“clinical* significant change”) OR TS=(“clinical* detectable* change”) OR TS=(“small detectable change”) OR TS=(“small real change”) OR TS=(“small detectable difference”) OR TS=(“small real difference”) OR TS=(“clinical* important* difference”) OR TS=(“clinical* significant difference”) OR TS=(“clinical* detectable* difference”) OR TS=(“clinical* important* change”) OR TS=(“meaningful change”) OR TS=(“Item response model”) OR TS=IRT OR TS=Rasch OR TS=(“Differential item functioning”) OR TS=DIF OR TS=(“Computer adaptive testing”) OR TS=(“item bank”) OR TS=(“cross-cultural equivalence”) 2. *name of desired outcome measure:* 3. 6?MWT OR 6?MWD OR 6?MW OR “six?min* walk* test*” OR 6?min* walk* test*” OR six?min* OR 6?min* OR 12?MWT OR 12?MWD OR 12?MW OR “twelve?min* walk* test*” OR 12?min* walk* test* OR twelve?min* OR 12?min* 4. ISWT OR ESWT OR “shuttle?walk*?test*” OR incremental?shuttle?walk*?test* OR endurance?shuttle?walk*?test* OR shuttle?walk* 5. stair*?climb* OR stair*?test* 6. “physical activity” OR “acceleromet*” OR “pedomet*” OR “actigraph*” OR “ambulatory activity” 7. “hand-held dynamomet$” OR “handheld dynamomet$” OR “hand held dynamomet$” OR “hand dynamomet$” OR “HHD” OR “dynamomet$” 8. “hand-grip dynamomet$” OR “hand grip dynamometry” OR “hand strength dynamomet$” OR “Jamar dynamomet$” OR “grip dynamomet$” OR “Takei Kiri Kogyo grip dynamomet$” OR “hand dynamomet$” OR “hand strength” OR (“grip AND strength”) 9. “manual muscle test$” OR “manual strength test” OR “Daniels and Worthingham’s muscle test$” OR “MMT” OR “muscle strength grad* scale*” OR “oxford muscle scale” OR “oxford muscle grad* scale” OR “oxford scale” OR [(muscle OR strength) AND (MRC OR “medical research council’)] 10. “1RM” OR “one-rep* max*” OR “one rep* max*” 11. “sit-to-stand” OR “sit to stand” OR “chair-stand test” OR “chair stand test” 12. (lung OR pulmonary OR thoracic) AND (cancer OR malignanc* OR oncology OR tumour OR resection) 13. #1 and #2 and #3 |

*Abbreviations: CINAHL, Cumulative Index to Nursing and Allied Health Literature; EMBASE, the Excerpta Medica Database; MESH, Medical Subject Heading Indexing.*
