# Supplementary material for: Functional capacity, physical activity and muscle strength assessment of individuals with non-small cell lung cancer: a systematic review of instruments and their measurement properties
Source: BMC Cancer. 2013 Mar 20;13:135. doi: 10.1186/1471-2407-13-135 (PMC3623892; doi:10.1186/1471-2407-13-135)
Supplement: Additional file 3 — Interpretability. Abbreviations: 6MWT, six minute-walk test; acc, accelerations; chemo, chemotherapy; CST, chair-stand test; E1, examiner one; E2, examiner two; Elb, elbow; E, extension; ECOG, Eastern Cooperative Oncology Group; ft, feet; gp, group; HGS, hand grip strength; hrs, hours; inpt, inpatients; IQR, inter-quartile range; ISWT, incremental-shuttle walk test; kg, kilogram; lbs, pounds; m, meters; MIC, minimal important change; min, minutes; ml, millilitres; N, newtons; outpt, outpatient; O2desat, oxygen desaturation; POC, post-operative complication; post-op, post-operative; pre-op, pre-operative; PS, performance status; RT, radiotherapy; s, seconds; SCT, stair-climb test; SD, standard deviation; SDD, smallest detectable difference; VO2peak, peak oxygen consumption; yr, year published.* results presented from most recent publication. [file 1471-2407-13-135-S3.docx]

Additional file 3: Inter-rater reliability, intra-rater reliability and measurement error associated with outcome measures

| **Author, year** | **Type of reliability and OM** | **Time interval** | **Reliability coefficient** | **Measurement error** |
| --- | --- | --- | --- | --- |
| **Muscle strength** | |  |  |  |
| Trutschnigg 2008 [28] | Intra-rater HGD | NR b/t tests | Jamar %CV repeated measures = 6.3, mean diff -0.03, p = 0.97  Biodex %CV repeated measures = 16.7, mean diff -2.47, p = 0.76 |  |
| Knols  2002 [52] | Inter-rater HHD pull gauge  1.Elb E  2.Knee E | 30min b/t examiners | 1. ICC = 0.90 (95%CI 0.81-0.94)  2. ICC = 0.96 (95%CI 0.92-0.98) | 1. SEM = 10.6 (95%CI -10.2 -31.4)  2. SEM = 19.8 (95%CI -19.0 - 58.6) |

*Abbreviations: 95%CI, 95% confidence intervals; %CV, percent coefficient of variation; b/t, between; E, extension; Elb, elbow; HGD, hand-grip dynamometry; HHD, hand-held dynamometry; ICC, intraclass correlation coefficient; mean diff, mean difference for repeated measures; min, minutes, OM, outcome measure; NR, not reported; SEM, standard error of measurement.*
