# Supplementary material for: Functional capacity, physical activity and muscle strength assessment of individuals with non-small cell lung cancer: a systematic review of instruments and their measurement properties
Source: BMC Cancer. 2013 Mar 20;13:135. doi: 10.1186/1471-2407-13-135 (PMC3623892; doi:10.1186/1471-2407-13-135)
Supplement: Additional file 4 — Inter-rater reliability, intra-rater reliability and measurement error associated with outcome measures. Abbreviations: 95% CI, 95% confidence intervals;%CV, percent coefficient of variation; b/t, between; E, extension; Elb, elbow; HGD, hand-grip dynamometry; HHD, hand-held dynamometry; ICC, intraclass correlation coefficient; mean diff, mean difference for repeated measures; min, minutes, OM, outcome measure; NR, not reported; SEM, standard error of measurement. [file 1471-2407-13-135-S4.doc]

Additional file 4: Interpretability

| **Author, yr** | **Mean (SD) scores** | | **Floor/ceiling effects present** | **Subgroup scores** | **MIC determined** |
| --- | --- | --- | --- | --- | --- |
| **Functional capacity** | | |  |  |  |
| Jones  2012 [40] | | Mean 6MWT = 396±116m |  |  |  |
| Pancieri  2010 [22] | | Mean 6MWT pre-op = 604±63m and 3month post-op = 599±74m p<0.001;  Mean SCT duration pre-op = 32.9±7.6seconds and 3month post-op = 33.7±8.5seconds p<0.001 |  |  |  |
| Kasmjanova 2009 [25] | | Mean initial 6MWT = 424±94m; mean pre-chemo 6MWT = 447±101m |  |  |  |
| Mao 2007*; Miller 2005 [26-27] | | Mean pre-RT 6MWT = 1396ft (range 300-2040)ft |  |  |  |
| Saad  2007 [24] | | Mean pre-op 6MWT = 509±99.5m; 6months post-op 6MWT = 506±95m |  |  |  |
| Parsons  2003 [39] | | Pre-op mean 6MWT = 390.8±105.14m |  |  |  |
| Pierce  1994 [23] | | Mean pre-op 6MWT = 537±85.3m |  | No POC mean 6MWT = 556±88m; POC mean 6MWT = 501±47m, p = 0.034 |  |
| Holden  1992 [41] | |  |  | Death <90days post-op mean 6MWT = 878±372ft and stairs climbed = 42±24; no or minor POC mean 6MWT = 1315±187ft and stairs climbed = 71±23 |  |
| England  2012 [44] | | Median ISWT = 320 [IQR 250-430]m; mean time mean = 358±95seconds |  |  |  |
| Win  2006 [30] | | Mean pre-op ISWT = 403±143.3m |  |  |  |
| Win  2004 [64] | | Mean pre-op ISWT = 395 (range 145-780)m |  | Pre-op ISWT <250m 50% females, 83% males poor post-op outcome;  Pre-op ISWT <400m 27% females, 43% males poor post-op outcome |  |
| Brunelli  2012 [43] | | Pre-op mean SCT altitude = 20±4.3m |  |  |  |
| Brunelli  2010 [31] | | Pre-op mean SCT altitude = 19±4.7m and speed of ascent = 10±2.4m/min |  |  |  |
| Brunelli  2008a [34] | | Pre-op mean SCT altitude = 19.5±4.6m |  | Mean altitude: no POC = 19.9±4.5m vs POC = 18.1±4.9m, p = 0.001 |  |
| Brunelli  2008b [33] | | Pre-op mean SCT altitude = 19.0±4.7m |  | Mean altitude: no POC = 19.8±4.5m vs POC = 18.3±4.8m, p = 0.003 |  |
| Koegelenberg 2008 [29] | | Pre-op mean SCT duration = 81.4±22.7seconds and speed of ascent = 15.49±3.32m/min | 43/44 patients climbed to max elevation of 20m |  |  |
| Nikolic  2008 [36] | | Pre-op mean SCT duration = 91±17seconds and stairs climbed = 88±12 |  | Mean SCT duration pre-op lobectomy = 91.9±15.9seconds; pre-op other surgery (pneumonectomy, bilobectomy, exploratory) = 89.4±19.1seconds, p = 0.48  Mean stairs climbed pre-op lobectomy = 91.0±5.8; pre-op other = 83.8±15.8, p = 0.003 |  |
| Brunelli  2007 [20] | |  |  | Mean VO_2_peak in pre-op SCT: lobectomy gp 26.7±3.8 ml/kg/min, 3months post-op 26.0±4.6ml/kg/min; pneumonectomy gp pre-op 26.1±2.4ml/kg/min, 3months post-op 23.2±3.3ml/kg/min |  |
| Toker  2007 [37] | | Pre-op mean SCT duration = 20.13±6.53seconds |  | No POC: pre-op mean SCT duration = 19.65±6.23seconds  POC: pre-op mean SCT duration = 21.67±6.55seconds |  |
| Brunelli  2005 [42] | | NR |  |  |  |
| Brunelli  2004 [35] | | Pre-op mean SCT duration = 115.1±30.6seconds |  | Mean altitude: no POC = 19.61±4.4m vs POC = 16.23±3.8m, p = 0.0004 |  |
| Brunelli  2003 [21] | |  |  | Mean altitude pre-op SCT: no O_2_ desat during post-op SCT = 20.01±4.7m vs O_2_ desat during post-op SCT = 20.51±4.5m, p = 0.6 |  |
| Brunelli  2002 [32] | | Pre-op mean SCT duration = 114.1±28.1seconds |  | Mean altitude pre-op SCT: no POC = 20.6±4.62m vs POC = 14.96±5.5m, p<0.0001 |  |
| Pate  1996 [38] | | Pre-op mean altitude = 19.0±4.8m, steps = 109±27, flights = 5.1±1.5 |  | No POC: pre-op mean altitude = 20.2±2.1m, steps = 115±12, flights = 5.5 ±0.6  POC: pre-op mean altitude = 18.3±5.9m, steps = 105±33, flights = 4.9 ±1.8 |  |
| **Physical activity** | | |  |  |  |
| Maddocks 2012 [47] | Mean daily step count = 4246±2983steps;  Mean daily time spent stepping = 1.1±0.7hrs | |  | Mean daily step count ECOG PS 0 = 8126±3334steps; PS 1 = 3791±2064steps; PS 2 = 2307±1518steps  Mean daily time spent stepping ECOG PS 0 = 1.9±0.7hrs; PS 1= 1.0±0.5hrs; PS 2 = 0.6±0.4hrs |  |
| Grutsch 2011a, 2011b; Du-Quiton 2010 [48-50] | Mean activity = 126.9±4.9acc/min | |  | Mean activity inpt = 111.7±71acc/min; outpt = 143.9±5.6acc/min p<0.05; healthy = 182.6±25acc/min |  |
| Novoa  2011 [51] | Mean step count = 8653.6±4578.3steps | |  |  |  |
| Maddocks 2010 [46] | Mean daily step count = 4244±2939 | |  |  |  |
| **Muscle strength** | | |  |  |  |
| Trutschnigg 2008 [28] | Jamar HGS mean = 68.4±23.6lbs | |  | Mean Jamar HGS males = 78.5±21.6lbs; females = 49.7±13.5lbs |  |
| Brown  2005 [53] |  | |  | Median (range) CST healthy = 0.97 (0.81-1.60)seconds; cancer = 1.50 (0.98-6.06)seconds |  |
| Knols  2002 [52] | Elb E E1 = 114.5±34.6N; E2 = 106.1±32.4N;  Knee E E1 = 280.5±91.9N; E2 = 275.5±95.2N | |  |  | Elb E SDD 29.4N  Knee E SDD 54.8N |

*Abbreviations: 6MWT, six minute-walk test; acc, accelerations; chemo, chemotherapy; CST, chair-stand test; E1, examiner one; E2, examiner two; Elb, elbow; E, extension; ECOG, Eastern Cooperative Oncology Group; ft, feet; gp, group; HGS, hand grip strength; hrs, hours; inpt, inpatients; IQR, inter-quartile range; ISWT, incremental-shuttle walk test; kg, kilogram; lbs, pounds; m, meters; MIC, minimal important change; min, minutes; ml, millilitres; N, newtons; outpt, outpatient; O_2_desat, oxygen desaturation; POC, post-operative complication; post-op, post-operative; pre-op, pre-operative; PS, performance status; RT, radiotherapy; s, seconds; SCT, stair-climb test; SD, standard deviation; SDD, smallest detectable difference; VO_2_peak, peak oxygen consumption; yr, year published.*

** results presented from most recent publication*
